# Bulked Segregant RNA-Seq Reveals Different Gene Expression Patterns and Mutant Genes Associated with the Zigzag Pattern of Tea Plants (*Camellia sinensis*)

**DOI:** 10.3390/ijms25084549

**Published:** 2024-04-21

**Authors:** Yuan-Yuan Ye, Ding-Ding Liu, Rong-Jin Tang, Yang Gong, Chen-Yu Zhang, Piao Mei, Chun-Lei Ma, Jie-Dan Chen

**Affiliations:** Key Laboratory of Biology, Genetics and Breeding of Special Economic Animals and Plants, Ministry of Agriculture and Rural Affairs, Tea Research Institute of the Chinese Academy of Agricultural Sciences, Hangzhou 310008, China; yeyuanyuan@tricaas.com (Y.-Y.Y.); liudingding@tricaas.com (D.-D.L.); tangrongjin@tricaas.com (R.-J.T.); gongyang@tricaas.com (Y.G.); zhangchenyu@tricaas.com (C.-Y.Z.); 82101235209@caas.cn (P.M.)

**Keywords:** zigzag-patterned stem, *Camellia sinensis*, BSR-seq, secondary cell wall, vascular bundle

## Abstract

The unique zigzag-patterned tea plant is a rare germplasm resource. However, the molecular mechanism behind the formation of zigzag stems remains unclear. To address this, a BC1 genetic population of tea plants with zigzag stems was studied using histological observation and bulked segregant RNA-seq. The analysis revealed 1494 differentially expressed genes (DEGs) between the upright and zigzag stem groups. These DEGs may regulate the transduction and biosynthesis of plant hormones, and the effects on the phenylpropane biosynthesis pathways may cause the accumulation of lignin. Tissue sections further supported this finding, showing differences in cell wall thickness between upright and curved stems, potentially due to lignin accumulation. Additionally, 262 single-nucleotide polymorphisms (SNPs) across 38 genes were identified as key SNPs, and 5 genes related to zigzag stems were identified through homologous gene function annotation. Mutations in these genes may impact auxin distribution and content, resulting in the asymmetric development of vascular bundles in curved stems. In summary, we identified the key genes associated with the tortuous phenotype by using BSR-seq on a BC1 population to minimize genetic background noise.

## 1. Introduction

Plant stems have many forms, including upright stems, pendulous stems, and tortuous stems; different stem forms enable plants to adapt to different external environments [1]. Tortuous stems twist naturally as they grow and have a dynamic beauty, which gives the plants unusual shapes and high ornamental value. Some plants exhibit a typical twisting of their stems, such as contorted *Corylus* [2], flexural willow [3], crooked plum [4], twisted jujube [5], and so on. Tortuous stems are often accompanied by plant height reduction and leaf shrinkage. They are generally curved, forming a zigzag pattern with leaf growth at the nodes but straight sections in the internodes [6]. Compared to vertical stems, the common anatomical manifestation of curved stems is abnormal vascular tissue development in different plants, such as an unusual cell size and form in the vascular bundle tissue [3,7,8] and the asymmetric development of vascular bundles [8].

The molecular mechanisms of tortuous stem formation are often studied using mutants. It has been found that the abnormal development of vascular bundles in curved stems is mostly influenced by the content and distribution of plant hormones. For example, the auxin mutants *A. thaliana axr1* and *lop1* exhibit tortuous stem traits [9,10], and gibberellin may regulate the formation of tendrils [11]. On the other hand, jasmonic acid may regulate the entanglement of tendril stems [12], and the enhancement of brassinolide signal transduction could result in the “S” curve of *Arabidopsis* stems [13]. The application of exogenous plant hormones on curved soybean [14] and curved *Brassica napus* rape [15] could restore the upright shape of the zigzag-shaped stems to a certain extent. A few key transcription factors can also affect secondary plant growth. For instance, D-type cyclins (CYCDs) can regulate cell division and vascular differentiation [16] and lead to the wavy-stem phenotype through the overexpression of *AtCYCD3;1* or *PtaCYCD1;2* [17,18]. Variations in genes belonging to the HD-ZIP III protein family could cause stem bending by affecting radial patterning in *Arabidopsis* [19], and mutations in the microRNA target of *popREV* and *SlREV* might have a similar result [20,21].

The tea plant (*Camellia sinensis*), originating from China, is an extremely important beverage crop cultivated in about 60 countries for its economic value. It is consumed all over the world due to its good taste and health functions [22,23]. The tea germplasm resources in China are quite abundant, with approximately 3000 resources available in the National Germplasm Hangzhou Tea Repository. The repository also includes the zigzag-shaped varieties, such as ‘Qiqv’, ‘Lianyuanqiqv’, ‘Longqv1hao’, and ‘Longqv2hao’ [24,25]. Recently, studies on tea plants have been focusing on their quality components [26,27,28] and stress resistance [29,30], while the morphological aspect is also under consideration.

Bulked segregant RNA-seq (BSR-seq) is an efficient method for exploring the mutations and expression patterns of genes related to specific traits in species with complex genomes [31]. BSR-seq is widely used in plant research [32,33], including tea plant research [34,35]. In this study, BSR-seq was performed on upright-stem and zigzag-stem plants to uncover the molecular regulatory mechanism underlying the formation of zigzag stems. This research provides novel transcriptomic evidence for the zigzag bending phenomenon in tea plants using advanced techniques.

## 2. Results

### 2.1. Phenotypic Characterization and Stem Tissue Sections of Erect and Zigzag-Shaped Shoots in Tea Plants

**Figure 1:**
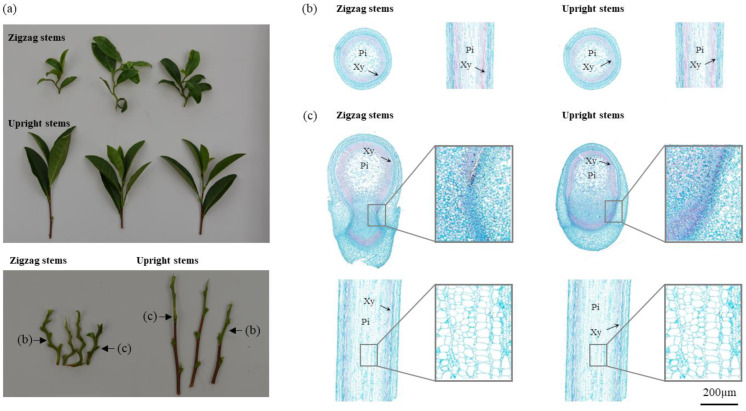
Phenotypic characterization and stem tissue sections of the BC1 population: (**a**) phenotype of the stem of upright and zigzag individuals; (**b**) transverse and longitudinal sections of the internode tissue; (**c**) transverse and longitudinal sections of the leaf axil (node) tissue.

Compared to other upright varieties, ‘Qiqv’ exhibits a distinctly curved stem that grows in a zigzag manner under natural conditions. We constructed the first generation of backcross offspring of ‘Qiqv’, using it as the male parent; the upright-type offspring resulting from a natural cross was used as the female parent. This led to the segregation of two distinct phenotypes: upright stem (‘UR’) and zigzag stem (‘ZZ’), with 41 individuals displaying the upright phenotype and 17 individuals displaying the zigzag phenotype. The zigzag phenotype was mainly characterized by outward-bending leaf axils and erect internodes. Additionally, the leaves of ‘UR’ plants were mostly folded inwards with wrinkled edges, while those of ‘ZZ’ plants were relatively flat (Figure 1a).

To further analyze the characteristics of the ‘ZZ’ and ‘UR’ offspring, we observed both transverse and longitudinal tissue sections of the node (leaf axils) and internode parts. The stem of tea plants mainly consists of epidermis, cortex, phloem, cambium, xylem, and pith. Compared to the erect shoots, generally, each tissue section of the zigzag shoots had a darker color and a more distinct cell wall, indicating a thicker secondary cell wall. There was little difference in the internode sections between ‘ZZ’ and ‘UR’ plants except for the xylem of ‘ZZ’ plants being thicker than that of ‘UR’ (Figure 1b). Regarding the axillary sections, the ‘ZZ’ plants had an obvious abnormal histological morphology: in the transverse section, the ‘UR’ shoot had an invagination inside the epidermal tissue for lateral bud growth, while the degree of invagination of the ‘ZZ’ shoot was very small, and the xylem and its surrounding tissue area was smaller; on the other hand, the pith cells of ‘ZZ’ plants were smaller in the longitudinal section, and the cell layers were greater in number and denser (Figure 1c).

### 2.2. RNA Sequencing and Reference Genome Alignment

Two expression libraries of the bulked groups of the plants with upright stems (‘UR-bg’) and plants with zigzag stems (‘ZZ-bg’) in the BC1 population were sequenced, thereby generating 11.62 Gb and 11.98 Gb raw data, respectively. After filtering adapters and low-quality sequences, 10.68 Gb and 11.08 Gb clean data, with Q30 at 90.73% and 91.21% and a GC content of 44.24% and 44.33%, were obtained. Then, the clean data were aligned to the reference genome ‘Shuchazao’ [36]. The total mapped rates were 98.11% and 98.39%, respectively (Table S1).

### 2.3. DEG Identification and Functional Enrichment Analysis

To understand the mechanism responsible for the formation of the zigzag stem of ‘Qiqv’, we applied bulked segregant RNA-seq (BSR-seq) to the progeny of the upright and zigzag plants via cluster separation analysis. The DEGs were then determined according to various parameters, with a *p*-value of ≤0.05.

**Figure 2:**
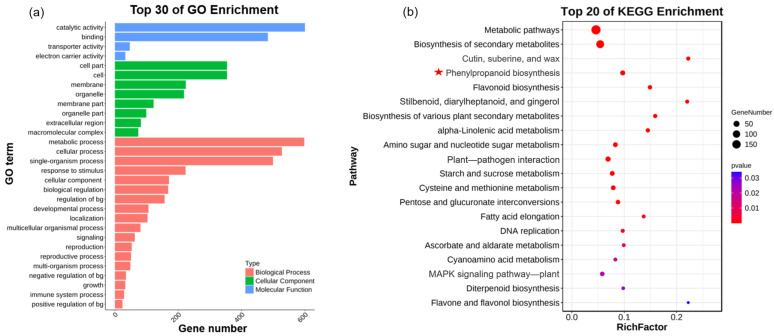
GO and KEGG enrichment analyses of the differentially expressed genes (DEGs) between the upright and zigzag bulked groups: (**a**) GO enrichment analysis and (**b**) KEGG enrichment analysis. The red star indicates the important metabolic pathway enriched for phenylpropane metabolism.

Between ‘UR-bg’ and ‘ZZ-bg’, there were 1494 DEGs (including 1232 upregulated and 262 downregulated DEGs). GO enrichment analyses demonstrated that the enriched terms containing the most DEGs were catalytic activity, metabolic process, cellular process, single-organism process, binding, cell, cell part, and membrane (Figure 2a). KEGG pathway enrichment analyses revealed that metabolic pathways; biosynthesis of secondary metabolites; cutin, suberine, and wax biosynthesis; phenylpropanoid biosynthesis; flavonoid biosynthesis; stilbenoid, diarylheptanoid, and gingerol biosynthesis; biosynthesis of various plant secondary metabolites; alpha-linolenic acid metabolism; amino sugar and nucleotide sugar metabolism; plant–pathogen interaction; starch and sucrose metabolism; cysteine and methionine metabolism; pentose and glucuronate interconversions; fatty acid elongation; DNA replication; and ascorbate and aldarate metabolism were all significantly (*p*-value ≤ 0.01) enriched (Figure 2b).

### 2.4. SNP Identification and Analysis of the Candidate Gene of Tortuous Stem

**Figure 3:**
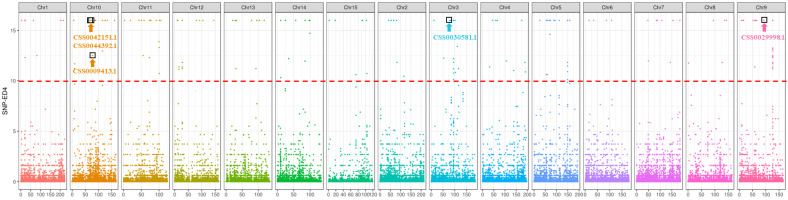
Distribution of the correlation thresholds of the Euclidean distance (ED) algorithm used to examine the chromosome of *Camellia sinensis*. The abscissa is the chromosome name, and the ordinate is the ED value; the colored dots represent the ED value of each SNP locus on the chromosome, the red dashed line represents the fitted ED value, and the points marked with boxes and arrows represent candidate genes.

A total of 505,579 high-quality and highly credible SNPs were finally obtained after detection using the genome analysis toolkit GATK [37] and filtering based on certain criteria. Then, the SNPs were used for the Euclidean distance (ED) analysis of the located target genes associated with the zigzag stem. According to the correlation threshold (ED^8^ > 10), a total of 262 SNP sites associated with the zigzag stem were identified via an ED algorithm analysis (Figure 3), and a total of 38 genes were annotated based on 69 of their SNPs (among which 47 SNPs were nonsynonymous) (Table S2). The five genes that may be related to the zigzag traits were selected according to the functional annotation of the genes (Table 1). CSS0027659.1 (homologous to *AtAMP1: Altered meristem program1*) may encode a glutamate carboxypeptidase that plays an important role in the development of the shoot’s apical meristem as well as phytohormone homeostasis [38]. CSS0030581.1 (homologous to *AtAroE: shikimate dehydrogenase AroE*) and CSS0032464.1 (homologous to *AtADT1: arogenate dehydratase*) are upstream of phenylpropanoid biosynthesis, and ADT1 plays a key role in catalyzing the conversion of arogenate to phenylalanine [39,40]. CSS0042151.1 (homologous to *AtAPM1: Aminopeptidase M1*) may negatively regulate PIN auxin transport proteins [41]. CSS0044392.1 (homologous to *AtREV: REVOLUTA*) may modulate interfascicular fiber and secondary xylem differentiation as well as determine vascular patterning and organ polarity [42,43]. These genes may affect the formation of the zigzag stem by regulating hormone transport and the differentiation of vascular tissues.

### 2.5. Analysis of Pathways of and Genes Related to Phenylpropane Biosynthesis

**Figure 4:**
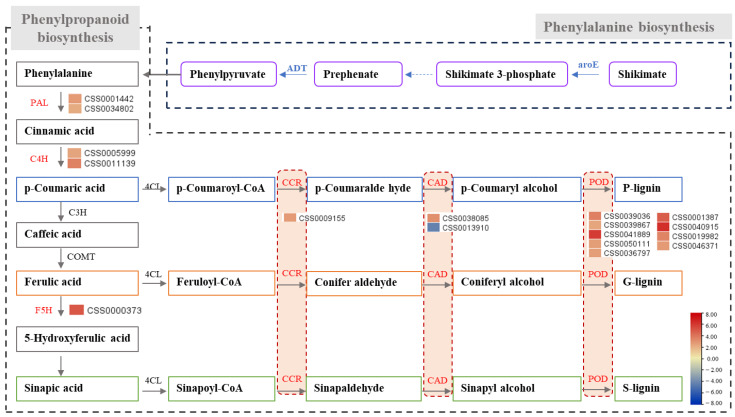
Pathways of and genes related to phenylpropane biosynthesis. The heatmap shows the log2FC of the differentially expressed genes (DEGs) between the zigzag and upright bulked groups. ADT1, arogenate dehydratase; AroE, shikimate dehydrogenase AroE; PAL, phenylalanine ammonia-lyase; C4H, cinnamic acid 4hydroxylase; F5H, ferulate 5−hydroxylase; CCR, Cinnamoyl CoA reductase; CAD, cinnamyl alcohol dehydrogenase; POD, peroxidase.

Phenylpropane is an important plant secondary metabolite and is the source of many metabolites such as flavonoids, lignin, lignan, cinnamic acid amide, and others. It plays a key role in plant development and plant–environment interactions [44]. In this study, the phenylpropane biosynthesis pathway was significantly enriched (Figure 4). The genes encoding phenylalanine ammonia-lyase (PAL) and cinnamic acid 4hydroxylase (C4H) were found to be significantly upregulated in ‘ZZ-bg’. PAL can direct more metabolic flux to the various branches of phenylpropanoid biosynthesis by catalyzing the formation of trans-cinnamic acid from phenylalanine [45]. C4H is related to lignin content, as evinced by the disruption of the C4H-encoding gene leading to a significant decrease in lignin content in *Arabidopsis* [46]. Furthermore, we observed that most of the DEGs encoding enzymes in the lignin biosynthesis branch pathway were also upregulated. In addition, there are two target genes encoding aroDE and ADT in the phenylalanine biosynthetic pathway that may also affect metabolic flux to phenylalanine.

### 2.6. Analysis of Plant Hormone Signaling Pathways and Related Genes

**Figure 5:**
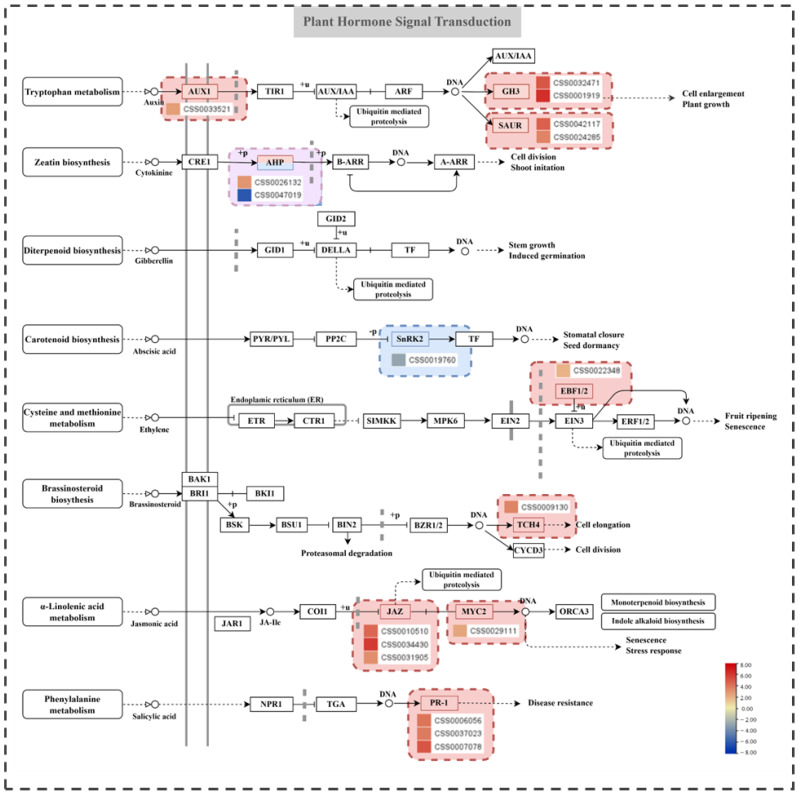
Plant hormone signaling pathways and related DEGs. The heatmap shows the log2FC of the differentially expressed genes (DEGs) between the zigzag and upright bulked groups.

Phytohormones’ distribution and content are closely related to the morphogenesis of plants; they regulate the activity of the meristem to establish an above-ground plant system by integrating signals from the environment with developmental stages and genetic factors [47]. In this study, seventeen DEGs existed in the signal transduction of auxin, cytokinine, abscisic acid, ethylene, brassinosteroid, jasmonic acid, and salicylic acid. Except for the two genes encoding SNF1-regulated protein kinase 2s (SnRK2s) and histidine-containing phosphotransfer protein (AHP), which were downregulated in the cytokinine and abscisic acid signaling pathways, the other genes were upregulated (Figure 5). Most DEGs were enriched in the auxin and JAZ signaling pathways. In auxin, the five DEGs encoding auxin transporter protein 1 (AUX1), indole-3-acetic acid-amido synthetase (GH3), and small auxin-up RNA (SAUR) were upregulated; they may regulate plants’ morphological development, thereby influencing auxin polar transport and signal response. In jasmonic acid, the four DEGs encoding jasmonate ZIM-domain (JAZ) and myelocytomatosis 2 (MYC2) were upregulated, which may affect resistance. In the brassinosteroid signaling pathway, one DEG encoding xyloglucosyl transferase (XTH) was upregulated and appeared to regulate the elongation of cells.

### 2.7. Gene Expression Validation through Quantitative Real-Time PCR

**Figure 6:**
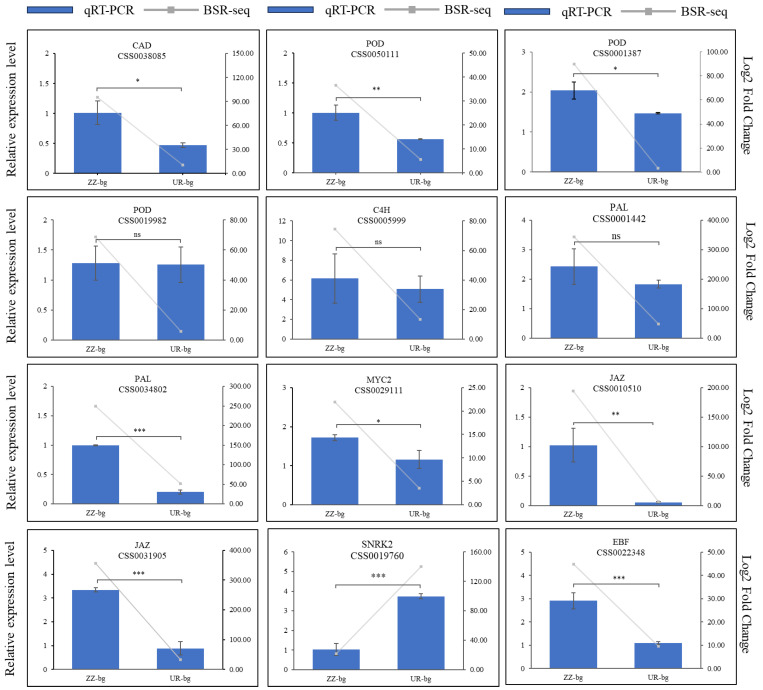
Validation of the relative expression levels of genes using quantitative RT-PCR (qRT-PCR). The bars represent the relative expression levels in the qRT-PCR, and the grey lines represent the log_2_ fold changes in the BSR-seq. qRT-PCR results are presented as the means (±SDs) of three technical replicates, utilizing the *CsGADPH* gene as a control. “ns” indicates no significant difference; * indicates a significant difference at the 0.01 level (*p* < 0.05); ** indicates a significant difference at the 0.01 level (*p* < 0.01); and *** indicates a significant difference at the 0.001 level (*p* < 0.001).

Twelve genes were randomly selected from the DEGs contained in the phenylpropane biosynthesis pathways and plant hormone signaling pathways; they were examined via qRT-PCR to validate the reliability of the RNA-seq results. The relative expression of eleven genes in ‘ZZ-bg’ was higher than that in ‘UR-bg’, and only that of one gene in ‘ZZ-bg’ was lower (Figure 6).

## 3. Discussion

The development of a zigzag stem is an unusual occurrence in tea plants, and ‘Qiqv’ is one of the few to feature a zigzag stem. This zigzag shape gives the tea plant a remarkable appearance, enhancing the ornamental value and aesthetic appeal of the crop, and therefore making its use in landscaping and other creative fields a real possibility. In this study, to focus on this zigzag characteristics, BC1 offsprings of ‘Qiqv’ both with upright and zigzag stems were taken as our research object, and BSR was used to investigate the mechanism responsible for the formation of the zigzag stem at the transcriptional level.

The phenotypic observation has demonstrated that the zigzag stems are composed of straight parts and bending parts. The straight parts are in the internode, while the bending parts are in the axils of the leaves and bend outward in the direction of leaf growth. Furthermore, to analyze the physiological causes of the zigzag stem, the ultrastructure of the two main parts of the ‘ZZ’ and ‘UR’ individuals were observed by both transverse and longitudinal cutting methods. It was found that the thickened secondary cell walls could be observed in all types of sections of the ‘ZZ’ individuals. The internode (upright part) has a thickened xylem and quite loosely spaced pith cells, which is similar to Cao’s observation on the zigzag-stem tea plant [48] and the tortuous-branched *Prunus mume* [4]. The tissue morphology of the bending site was distinctly different between the ‘ZZ’ and ‘UR’ individuals. The proportion of the xylem area was decreased, and cells near the lateral organ were increased and expanded to the periphery. Pith cells were smaller but higher in number, which was different from the bending parts. The phenotypic and anatomical observations indicated that the zigzag stems had thicker cell walls; the bending parts may be the underlying reason for the formation of the zigzag stem, and the tissue morphology of the bending site was abnormally regulated.

Our analysis of the pathways of phenylpropanoid biosynthesis revealed that lignin may be accumulated in ‘ZZ’ individuals. Lignin is a component of secondary cell walls [49], and its accumulation may lead to the thickening of secondary cell walls (as observed in the tissue sections). Thickened cell walls affect the formation of plant morphology, provide stronger mechanical support [50], and also increase plants’ capacity for resistance [51,52]. Among the significantly enriched pathways, cutin, suberine, and wax biosynthesis; flavonoid biosynthesis; and plant–pathogen interaction are also associated with resistance [53,54], and their DEGs are mostly upregulated in ‘ZZ-bg’. Phenylpropanoid biosynthesis and alpha-linolenic acid metabolism are the pathways through which SA and JA are synthesized; they play central roles in plants’ resistance to stress [55]. In the SA and JA signal transduction pathways, PR-1-, JAZ-, and MYC-encoding genes are upregulated. These pathways have been studied in soybean and *Brassica napus* [14,15]. In general, there is a balance between growth and resistance in organisms, and the ‘ZZ’ plants appeared to be weaker than the ‘UR’ plants in terms of growth. This may indicate that curved plants expend more energy in resisting stress than in growing. Accordingly, functional enrichment analysis revealed that the secondary cell walls of the curved single plants may be thickened and their resistance-related activities therefore enhanced.

Subsequently, we further analyzed the potential mechanisms involved in the regulation of zigzag stems. Previous studies have demonstrated that hormones may influence bending traits. Among them, auxin polar transport appears to greatly control the curvature of stems. Auxin is primarily synthesized in the shoot apex and developing leaf primordia before being transported to various tissues and organs through xylem parenchyma cells under the influence of transport carriers and forming an auxin concentration gradient [56,57]. This concentration gradient affects the formation and tropism of vascular bundles, which may result in the curvature of the stem [58,59]. The asymmetric distribution of auxin transport carriers is the main reason for its polar transport. The auxin efflux carrier PIN plays a major role in polar transport [60,61]. In our study, we found a mutant gene, *APM1,* that may negatively regulate auxin polar transport by PIN [41]. *Brassica napus stb1 mutant* was also found to influence the downregulation of *APM1*, which thus attests to the close association of *APM1* with the development of vascular tissue through participation in polar auxin transport [15]. Mutations in *APM1* may affect PINs’ effects on auxin polar transport and lead to the abnormal development of vascular bundles. Alongside PINs, AUX1, the auxin influx carrier, also plays a critical role in the polarity of auxin movement [62]. Additionally, the AUX1-encoding gene’s differential expression may also influence auxin polar transport [14]. In addition, auxin signal transductions may also affect the curvature of stems [15]. SAUR and GH3 were found to be upregulated in the ‘ZZ’ plants in our study. Hence, it seems that auxin transport and signal transduction may induce the abnormal development of vascular bundles, thereby causing the zigzag character of ‘Qiqv’.

Alongside hormones, the asymmetric development of vascular bundles caused by transcription factors also influences the formation of curved stems [6]. The HD-ZIP III family of transcription factors is essential for plants’ growth and response to the environment; they take effect by regulating the development of vascular bundles [63,64]. In this study, a nonsynonymous nucleotide substitution was identified as a gene homologous to the *Arabidopsis REV* gene, which encodes the homeobox-leucine zipper protein REVOLUTA. REVOLUTA plays a central role in regulating interfascicular fiber and secondary xylem differentiation and may be involved in the determination of vascular patterning and organ polarity in *Arabidopsis* [42,43,65]. Moreover, REVOLUTA also performs similar functions regarding plant growth and environmental response in other species [20,21,66]. Mutations in the regulatory binding sites of the *REV* gene can lead to the appearance of the curved stem phenotypic trait. Thus, the mutation of the *REV* homologous gene in tea plants may also contribute to the formation of a zigzag stem by affecting the formation of vascular tissues.

## 4. Materials and Methods

### 4.1. Plant Materials

‘Qiqv’ is a tea variety characterized by zigzag-shaped branches. A backcross population was constructed by using ‘Qiqv’ and its natural hybrids. The upright stem and zigzag stem characteristics were separated within the BC1 population. The non-lignified stem segments (including 3–4 internodes) were selected from 7 upright- and 7 curved-stem progenies and mixed for bulked segregant RNA sequencing. The BC1 population and their progenies were planted in the Shengzhou experimental field of the Tea Research Institute within the Chinese Academy of Agricultural Sciences (CAAS), using conventional and uniform horticultural practices. The samples were stored at −80 °C for the extraction of RNA.

### 4.2. Observation of Tissue Sections 

After soaking in water, the stems of the upright and zigzag plants were divided into two components: internodes and leaf attachment sites (Figure 1b,c). Tissue slices of approximately 3 mm thickness were cut horizontally and vertically from each part and placed in centrifuge tubes containing a fixative solution. To prevent floating, the slices were packed with absorbent cotton until they sank completely. After categorizing the tissue slices, they were placed in a dehydration box, sealed, and softened at a constant temperature. The softened tissues were then subjected to dehydration, embedding, sectioning, slide baking, and dewaxing. The staining process involved sequential steps with hematoxylin dye, gradient alcohol decolorization, fast green dye, and dehydration with anhydrous ethanol. After air-drying, the samples were placed in xylene for transparency treatment and mounted with neutral gum. Microscopic examination was performed using an optical microscope (NIKON ECLIPSE E100, Tokyo, Japan), and the images were captured and analyzed using an imaging system (NIKON DS-U3, Tokyo, Japan).

### 4.3. RNA Extraction, Library Construction, and RNA Sequencing

The total RNA from each sample was extracted using the EASYspin Plus Polysaccharide Polyphenol/Complex Plant RNA Rapid Extraction Kit (Beijing Edilai Biological Technology Co., Ltd., Beijing, China). After evaluating RNA purity and determining concentration using agarose gel electrophoresis and Nanodrop 2000 (Thermo Fisher Scientific, Waltham， MA, USA), respectively, the samples were sent to Shanghai Paisenno Biotechnology Co., Ltd. (Shanghai, China) for transcriptome sequencing. The total RNA was subjected to mRNA purification, mRNA fragmentation, reverse transcription, PCR enrichment, and library quality assessment to construct libraries suitable for high-throughput sequencing (with insert fragments of 400 bp and a concentration of 2 nM). The libraries were sequenced using the Illumina NovaSeq platform with a 2 × 150 bp paired-end sequencing strategy to obtain raw data. After filtering adaptors and low-quality reads using fastp [67], the obtained clean data were aligned to the reference genome ‘Shuchazao’ of the tea plant [36] using Burrows–Wheeler Alignment tool (BWA) [68], resulting in BAM files. Then, the BAM files were compared with the structural annotation file (GTF file) of the reference genome using HTSeq [69] for subsequent SNP calling, DEGs analysis, and gene functional annotation analysis.

### 4.4. SNP Calling, DEG Identification, and Functional Enrichment Analysis

The BAM files were then sorted and de-duplicated using picard 1.107, and GATK 4.40.0 software was used to detect SNP calling. Subsequently, SNPs that correlated strongly with target traits were screened out according to their ED value. Then, based on the functional annotation of genes containing these SNPs, genes that may have been related to the target traits were screened out. Gene expression levels were estimated using fragments per kilobase per million reads (FPKM). Differential expression analysis was performed using DESeq2 [70], and the differentially expressed genes (DEGs) were filtered with a *p*-value of < 0.05. Gene ontology (GO) and Kyoto Encyclopedia of Genes and Genome (KEGG) enrichment analyses were performed on the Gene Denovo Cloud platform (https://www.omicshare.com/, accessed on 9 November 2023). The heatmap of expression was drawn with TBtools 2.067 [71].

### 4.5. qRT-PCR Validation

qRT-PCR was implemented to validate the gene expression differences in 12 DEGs between ‘ZZ-bg’ and ‘UR-bg’. Total RNA samples were converted into cDNA via a reverse transcription reaction using a High-Capacity cDNA Reverse Transcription Kit (Applied Biosystems, Foster City, CA, USA). The primers designed for the 12 DEGs are listed in Table S3. qRT-PCR was performed using a LightCycler 480 System with LightCycler 480 SYBR Green I Master. The qPCR reaction system (10 μL) consisted of 5 μL of SYBR Green Master Mix, 0.5 μL of forward/reverse primer, 1 μL of cDNA, and 3 μL of sterile water. The analysis proceeded as follows: 10 min of initiation followed by 40 cycles of 94 °C for 10 s, 58 °C for 15 s, and 72 °C for 12 s. The results were calculated using the2^−ΔΔCT^ method with the *CsGADPH* gene as a control. Three technical replicates were prepared for each sample.

## 5. Conclusions

**Figure 7:**
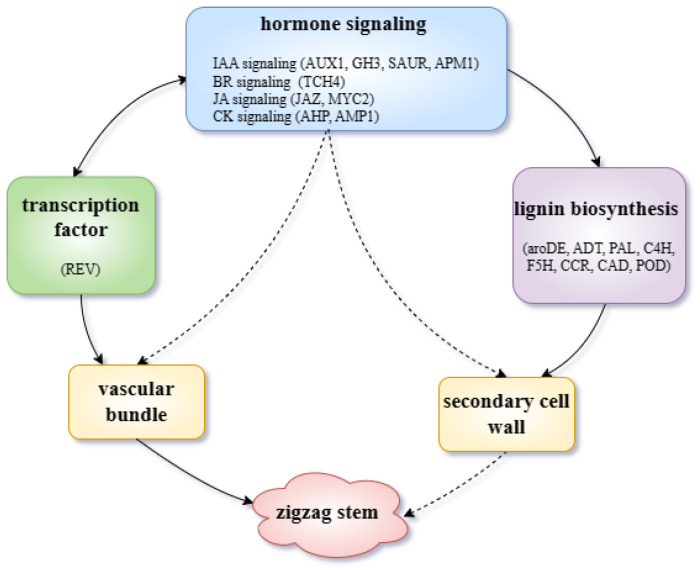
Conclusions regarding transcription-level regulation of the formation of zigzag stems in *Camellia sinensis*. The solid line represents direct regulation, and the dotted line represents indirect regulation.

In this study, we conducted observations of tissue sections and BSR-seq analyses of the BC1 population of the tea plant ‘Qiqv’. We observed that secondary cell walls were thicker and vascular bundle development proceeded abnormally in plants with zigzag stems (compared with upright-stemmed plants). The 1494 DEGs and 262 SNPs obtained through BSR-seq were then analyzed. We found that the upregulation of the expression of genes in the lignin biosynthesis pathway in the curved bulked groups may lead to the accumulation of lignin and consequent thickening of secondary walls. A variety of genes and DEGs related to auxin synthesis, transport, and signal transduction—as well as the mutation of the key transcription factor—may affect the abnormal expression of vascular bundles and result in stems exhibiting zigzag patterns (Figure 7). These findings present strong evidence that will further our collective understanding of the molecular mechanism responsible for the formation of zigzag stems in tea plants; they may thus open up new avenues for research into tea germplasm resources.

## Figures and Tables

**Table 1:** Target genes associated with the formation of a zigzag stem according to BSR-seq.

Gene_id	Function	Mutation Type
CSS0027659.1	regulating meristem function; balancing auxin signaling	synonymous
CSS0030581.1	shikimate dehydrogenase	nonsynonymous
CSS0009413.1	arogenate dehydratase/prephenate dehydratase	synonymous
CSS0042151.1	negative regulation of PIN auxin transport proteins	nonsynonymous
CSS0044392.1	regulation of interfascicular fiber and secondary xylem differentiation; determination of vascular patterning and organ polarity	nonsynonymous

## Data Availability

Data will be made available on request.

## Supplementary Materials

The following supporting information can be downloaded at: https://www.mdpi.com/article/10.3390/ijms25084549/s1.
